# CD133作为肺癌干细胞标记物的应用及其局限性

**DOI:** 10.3779/j.issn.1009-3419.2011.10.10

**Published:** 2011-10-20

**Affiliations:** 200092 上海，上海交通大学医学院附属新华医院心胸外科 Department of Thoracic and Cardiovascular Surgery, Xinhua Hospital, School of Medicine, Shanghai Jiaotong University, Shanghai 200092, China

**Keywords:** 干细胞, 肺肿瘤, CD133, Stem cells, Lung neoplasms, CD133

## Abstract

肺癌是临床上最常见的恶性肿瘤之一，尚缺乏预后较为理想的治疗方案。肿瘤干细胞学说认为肺癌干细胞是肺癌发生、发展、转移、复发、耐受放化疗以及肺癌细胞具有侵袭性的主要原因。近年来越来越多的机构利用CD133糖基化表位来鉴定、分离、提纯肺癌干细胞。然而，随着研究的深入，CD133作为肺癌干细胞标记物逐渐受到质疑。本文对近年来利用CD133作为肺癌干细胞表面标记物的应用及其局限性做一综述。

## Abstract (en)

Lung cancer is one of the most common tumor, which lacks of effective clinical treatment to lead to desirable prognosis. According to cancer stem cell hypothesis, lung cancer stem cells are considered to be responsible for carcinogenesis, development, metastasis, recurrence, invasion, resistance to chemotherapy and radiotherapy of lung cancer. In recent years, more and more institutes used glycosylated CD133 epitopes to define, isolate, purify lung cancer stem cells. However, along with deeply research, the application of CD133 epitopes in lung cancer stem cell research is questioned. The utilization and limitation of CD133 epitopes in lung cancer stem cells research for the past few years is summaried in this review.

肺癌是世界上最常见的恶性肿瘤之一，发病率及病死率呈明显增高的趋势。虽然近年来在肺癌的诊断和治疗上有了很大的进展，但是肺癌患者的5年生存率仍未超过16%^[1]^。肿瘤干细胞学说认为，肺癌组织中一类数量较少但具有较强的致瘤性、自我更新、无限增殖以及分化潜能的细胞是肺癌发生、发展、转移、复发、耐受放化疗以及肺癌细胞具有侵袭性的主要原因，因其具有干细胞样特性被称为肺癌干细胞。在肺癌组织中靶定并清除这类肿瘤干细胞或许会给肺癌的治疗带来更为有效的方案^[2]^。近年研究发现在人类多种实体肿瘤中存在CD133^+^肿瘤干细胞，如肺癌、室管膜瘤、前列腺癌、结肠癌、肝癌、喉癌、黑色素瘤、卵巢癌、胰腺癌^[3]^、骨肉瘤^[4]^及胆囊癌^[5]^等。随着在脑肿瘤干细胞中AC133/AC141阳性细胞群的发现，以AC133和AC141表位作为标记已经广泛应用于其他实质性肿瘤干细胞的纯化。有些机构联合应用AC133和AC141表位作为肿瘤干细胞的标记物。但已有研究^[6]^检测到AC133与AC141表位表达的不一致；并且即使能够检测到CD133蛋白的存在其表位也可能缺失^[3]^。此外，有研究^[7, 8]^显示CD133的表达受氧浓度水平调节。此外还未确定的CD133的生物学功能、是否存在CD133阴性的肺癌干细胞以及目前流式细胞术进行细胞分析时存在的潜在技术缺陷，提示若在实验中用CD133作为肺癌干细胞的标记物需要进行严密的评估以及慎重的考虑，并且要重视其它一些直接参与保持肺癌干细胞特性的肿瘤干细胞表面标记物。

## 1 干细胞与肿瘤干细胞

干细胞是一类具有自我更新、无限增殖和多向分化潜能的细胞。1959年Makino等^[9]^首次提出肿瘤干细胞假说，并指出肿瘤可能由肿瘤干细胞产生。直到2001年肿瘤干细胞这一概念才被正式提出。肿瘤干细胞学说认为肿瘤组织中存在一类数量较少，但是具有较强致瘤性，自我更新、无限增殖以及分化潜能的细胞，因其具有干细胞样特性被称为肿瘤干细胞（cancer stem cell, CSC）。这类细胞是肿瘤发生、发展、转移、复发、耐受放化疗以及肿瘤细胞具有侵袭性的主要原因。目前研究认为肿瘤干细胞与干细胞存在以下相似点：①均具有自我更新、无限增殖和分化潜能，不同的是干细胞的增殖具有稳定性，当组织处于稳定状态时，平均一个干细胞产生一个子代干细胞和一个特定分化细胞，其干细胞总数保持恒定，而肿瘤细胞无自稳定性的特点；②均存在相似的生长调控信号通路，如Wnt/β-catenin^[10]^、Notch、Shh（Sonichedgehog）和Bmi-1等信号通路^[11]^，它们的异常表达可引起细胞过度增殖而导致肿瘤发生；③具有端粒酶活性以及扩增的端粒重复序列，而人类终末分化体细胞不具有或者具有极低的端粒酶活性^[12]^；④具有比较类似的表面标志物，如lnestin、CDl33、sox-2、musashi-1、Bmi-l、survivin、ABCG-2等；⑤干细胞具有迁移的特性，而CSCs则具有转移的能力；⑥长期处于细胞增殖周期的静止期（G_0_）；⑦均通过对称及不对称两种方式分裂；⑧干细胞与CSCs在一定的条件下还可以相互转化，如胚胎植入体内可以诱导分化成畸胎瘤，而畸胎瘤细胞注入鼠囊胚内可以形成正常胚胎。

## 2 CD133的发现

人CD133蛋白与鼠prominin-1（一种富集于神经上皮干细胞顶端表面微绒毛上的表面蛋白）有大约60%的同源性^[13]^，故也称人CD133为人prominin-1。CD133是一个5次跨膜糖蛋白，最初于1997年作为CD34阳性造血干细胞上的表面抗原被AC133单抗靶定而被发现^[14]^。与此同时测定其分子量约为97 kDa，由865个氨基酸组成^[15]^，包括85个氨基酸N端胞外域、5个跨膜区、2个大的胞外环（包含8个潜在的连接N端的糖基化位点）以及一个由50个氨基酸组成的胞内尾端。有研究^[14]^表明AC133抗原是一个表观分子量为120 kDa的糖基化蛋白。同年发现了另一个CD133糖基化表位-AC141^[15]^。荧光激活细胞分选术（fluorescence-activated cell sorting, FACS）显示抗AC133抗体和抗AC141抗体能够识别空间结构特异的表位^[14]^。对经过衣霉素处理的细胞进行免疫沉淀试验发现AC133和AC141都能够识别糖基化的结构^[15]^，提示由抗AC141抗体所识别的CD133的糖基化表位与抗AC133抗体所识别的CD133的糖基化表位空间结构不一致，分别称为AC133表位和AC141表位。针对AC133表位和AC141表位开发出了商业化的单克隆抗体CD133-1和CD133-2，并被广泛用于CD133表达的检测^[3]^，可以认为AC133和AC141只是糖基化CD133表位的两个亚型。

## 3 CD133阳性的肺癌细胞

Eramo等^[16]^检测到肺癌组织中存在CD133阳性的肿瘤细胞，其数量较少，但与对照组的正常肺组织相比肿瘤中CD133表达的百分比总是高。选取的肿瘤组织类型包括鳞癌、腺癌、大细胞肺癌以及小细胞肺癌。进一步定量检测发现CD133表达百分比与肿瘤亚型无明显相关性。同时检测到CD133阳性的非小细胞肺癌表达上皮细胞粘附因子（Ep-CAM），与结肠癌相似^[17]^，表明肺癌中CD133阳性的肿瘤细胞与未分化的上皮细胞数量增多有关。Eramo等^[16]^将CD133阳性肺癌细胞和CD133阴性肺癌细胞从肺癌标本中分离出来分别移植入小鼠中，CD133阳性的肺癌细胞能够在小鼠中再生出原始肿瘤，但CD133阴性的肺癌细胞则缺乏这种能力。进一步量化将104个CD133阳性肺癌细胞经皮下注射移植入SCID小鼠可再生出原始肿瘤，但是105个CD133阴性肺癌细胞以同样的方式移植入同样的小鼠却未能再生出原始肿瘤，表明CD133阳性的肺癌细胞与CD133阴性的肺癌细胞相比具有更强的致瘤能力。与其他肿瘤中发现的致瘤细胞类似，CD133阳性的肺癌细胞较CD133阴性的肺癌细胞具有更强的增殖及自我更新潜能。分别将CD133阴性的肺癌细胞和CD133阳性的肺癌细胞在相同的适宜培养条件下进行体外培养，CD133阴性肺癌细胞在培养2周-3周后逐渐死亡，且未能获得CD133表位表达；1个-2个月后CD133阳性肺癌细胞形成肿瘤球并继续生长，检测肿瘤球发现其含有数量较多的自我更新细胞约为5%-30%。此外，CD133阳性肺癌细胞在一定的培养条件下能够生发出相应类型的肺癌细胞，经检测发现其表达谱系特异的标记物，但丢失了CD133的表达。

Bertolini等^[18]^也发现在原发非小细胞肺癌组织中CD133阳性细胞远比正常肺组织多，并且表现出干细胞样特性以及对于铂类药物的耐受。Chen等^[19]^从肺癌组织样本中分离得到CD133阳性肺癌细胞和CD133阴性细胞，发现CD133阳性的肺癌细胞高表达Oct4。Oct4表达于多能胚胎干细胞，并可以作为多能胚胎干细胞的一个重要标记物^[20-22]^。同时，Chen等^[19]^测得CD133阳性的肺癌细胞，高度共表达多重耐药标记ABCG2，并表现出较强的耐受放化疗特性。ABCG2最早发现于乳腺癌细胞株MCF-7/Adr-Vp3000中，并被认为与乳腺癌多耐药性相关^[23]^。目前已被证实多种肿瘤干细胞中表达ABCG2并与其耐药性相关。

近年来根据CD133阳性肺癌细胞存在的干细胞特性进行了较多的研究。Woo等^[24]^对177例Ⅰ期肺腺癌患者的样本分析发现，高表达CD133患者其5年无瘤生存率显著低于低表达CD133患者，并认为CD133阳性肺腺癌细胞表达率的高低可作为一个独立因子来评价患者术后肿瘤的复发情况。另有报道^[25]^称CD133与非小细胞肺癌耐药性相关，但是与患者生存期无明显关联。随着研究的深入或许在不久的将来CD133将成为临床上肺癌诊断、预后的重要指标。更为重要的是，若能够靶定CD133肺癌细胞并抑制其活性进而将其清除，或许将会给肺癌的临床治疗带来新的突破^[26]^。

## 4 CD133作为肺癌干细胞表面标记物的局限性

### 4.1 AC133和AC141作为肿瘤干细胞标记表位的争论

到目前为止，几乎所有CD133相关的实验都运用到抗AC133抗体和抗AC141抗体这两种单克隆抗体，其最初是用来纯化和标记造血干细胞及其前体细胞，后来用作鉴定CD133表面抗原^[14]^。虽然运用抗AC133和抗AC141单抗来纯化和鉴定肺癌干细胞群的成功报道越来越多，但是在解读这些结果的时候需要考虑以下几点：①目前已经证实AC133和AC141是两个空间结构不同的糖基化表位^[14, 15]^，但是AC133和AC141在分子水平上的属性还没有确切的证据，在CD133蛋白上被AC133、AC141单抗靶定的氨基酸残基的精确定位还没有十分明确^[3]^；②虽然AC133和AC141单抗常规用以分析以及提纯细胞，但是在骨髓增生异常综合征和急性髓系白血病患者中其骨髓前体细胞和外周血干细胞上的AC133和AC141表位表达不一致^[6]^。有研究^[27, 28]^表明AC133和AC141表位独立于CD133蛋白或其mRNA而表达下调，并且发现CD133蛋白的mRNA其组织分布的广度更甚于AC133表位的表达范围^[15]^；③CD133存在诸多亚型，人类CD133基因包括在4号染色体上至少152 kb长的37个外显子，迄今已有报道过人和鼠中存在数个CD133的亚型^[29-31]^，如一种CD133亚型（称为CD133-2）缺乏外显子4，导致其胞外域N端缺失9个氨基酸，但被认为是造血干细胞上主要的CD133亚型，并且推测其能够被AC133和AC141单抗所识别。AC133和AC141只是CD133蛋白的两个糖基化表位，目前对于缺失AC133和/或AC141表位的CD133亚型是否存在还是一个未知数，以及不能完全排除AC133/AC141抗体还可识别CD133以外其他分子上的糖基化表位的可能^[5]^。有研究^[28]^采用非糖基化依赖的CD133抗体，发现分化后的Caco2细胞仍然恒定表达CD133未明显降低。因此，没有经过确切检测CD133蛋白或mRNA水平而把AC133或AC141表位阴性的细胞称之为CD133阴性细胞不合理，将CD133的糖基化表位作为肿瘤干细胞的标记比CD133蛋白水平可能要更确切^[3]^；④AC141单抗与细胞角蛋白18存在交叉反应^[32]^，这对经过固定的细胞或者破坏了细胞膜的细胞进行免疫组化研究时将会是一个干扰因素^[3]^。

### 4.2 CD133的生物学功能尚不明确

目前对CD133或其糖基化表位在调节肿瘤干细胞表型时是否起着直接的作用还不清楚，对于CD133的生物学功能还知之甚少^[3]^。CD133位于各类细胞中质膜的突出部，其与膜部胆固醇相互作用，并且作为脂微域的标记物^[33]^。推测这些膜微域中富含维持干细胞特性的组分，一旦通过细胞的不对称分裂将其丢失，或许会促进细胞的分化^[34]^。有研究^[35]^证明单个核苷酸的缺失会造成CD133的缩短从而导致视网膜变性。迄今为止尚缺乏定向敲除CD133的研究，对AC133/AC141阳性的肺癌干细胞进行定向敲除CD133，然后检测其致瘤能力，这或许会在确定CD133是否在肺癌干细胞的致瘤性中扮演重要角色提供进一步的实验依据^[3]^。

### 4.3 CD133阴性的肺癌干细胞

因为存在很多影响CD133表达的因素，所以不能完全排除存在CD133阴性的肺癌干细胞的可能性。有多项研究^[36-39]^认为存在CD133阴性胶质瘤干细胞的可能性。董强刚等^[40]^检测了A549和SPC-A1肺腺癌细胞，未检测到CD133特异性mRNA，进一步采用流式细胞术也未检测到CD133蛋白表达，表明可能存在CD133阴性的肺癌干细胞。Meng等^[41]^检测A549和H445肺癌细胞发现，CD133阴性细胞和CD133阳性细胞一样具有成瘤、自我更新、增殖、分化、侵袭以及耐受化疗的特性，并认为A549和H446肺癌细胞中CD133阴性细胞和CD133阳性细胞均包含有肺癌起始细胞即肺癌干细胞。

### 4.4 氧浓度水平调节CD133的表达

有研究^[7, 8]^发现在培养髓母细胞瘤和神经胶质瘤细胞时降低给氧量（20%降至2%-3%）能够增加AC133和AC141的表达及CD133mRNA的水平，并且已经证明低氧能够促使CD133阳性的胰腺癌细胞更具侵袭性以及增殖能力^[42]^。在体外进行肿瘤干细胞培养时的氧浓度水平约在20%左右，肺癌在人肺组织中生长环境的氧浓度水平以及将CD133阳性肺癌细胞通过皮下注射移植入SCID鼠建立肿瘤再生模型时，其生长环境的氧浓度水平目前还没有一个确切的数据，可能会因为氧浓度水平的不同导致CD133的表达水平有所差异，并因此影响其致瘤潜能。因此，今后需要进一步的实验测定不同环境的氧浓度以及氧浓度在肿瘤干细胞试验中是否为一个重要的实验变量。

### 4.5 流式细胞术的局限性

目前流式细胞术广泛应用于检测CD133，被认为具有高精确性及高特异性^[43]^，但仍存在一定的局限性，包括以下几方面：①有研究^[15, 44]^称CD133的865个氨基酸的序列中存在79个胰酶切割位点，其中亦包括多个存在于CD133糖基化胞外域中的位点，胰酶消化分离细胞可能会影响CD133的形态结构和/或功能，从而影响其分选；②有报道^[44]^在胞浆中发现存在CD133蛋白，而这部分蛋白不会被流式细胞仪所检测出来，导致CD133的检出率下降；③有研究^[45]^认为经过流式细胞术分选的CD133阳性细胞在进行体外培养时会影响其以肿瘤球的形式生长；④对CD133表达程度的描述存在主观性。

综上所述，在当前肺癌干细胞的研究中CD133充当着十分重要的角色，但是CD133作为肺癌干细胞标记物仍存在诸多的疑问和未知。由于影响因素较多且较为复杂，甚至可能很多影响因素还未被意识到，当应用CD133对肺癌干细胞进行鉴定提纯时，要持有谨慎的态度。目前多种实体肿瘤已联合应用多个标记物来鉴定提纯肿瘤干细胞，这在当前的肺癌干细胞研究中亦不失为一种尝试。当然，寻找特异性的肺癌干细胞标记物和基因表达谱仍是今后努力的方向，并期望由此给临床肺癌的诊断和治疗开创新的局面。

## References

[1] Siegel R, Ward E, Brawley O (2011). Cancer statistics, 2011: the impact of eliminating socioeconomic and racial disparities on premature cancer deaths. CA Cancer J Clin.

[2] Reya T, Morrison SJ, Clarke MF (2001). Stem cells, cancer, and cancer stem cells. Nature.

[3] Bidlingmaier S, Zhu X, Liu B (2008). The utility and limitations of glycosylated human CD133 epitopes in defining cancel stem cells. J Mol Med.

[4] Tirino V, Desiderio V, Paino F (2011). Human primary bone sarcomas contain CD133^+^ cancer stem cells displaying high tumorigenicity *in vivo*. FASEB J.

[5] Shi CJ, Gao J, Wang M (2011). CD133(+) gallbladder carcinoma cells exhibit self-renewal ability and tumorigenicity. World J Gastroenterol.

[6] Green WB, Slovak ML, Chen IM (1999). Lack of IRF-1 expression in acute promyelocytic leukemia and in a subset of acute myeloid leukemias with del(5)(q31). Leukemia.

[7] Blazek ER, Foutch JL, Maki G (2007). Daoy medulloblastoma cells that express CD133 are radioresistant relative to CD133- cells, and the CD133+ sector is enlarged by hypoxia. Int J Radiat Oncol Biol Phys.

[8] Platet N, Liu SY, Atifi ME (2007). Influence of oxygen tension on CD133 phenotype in human glioma cell cultures. Cancer Lett.

[9] Makino S (1959). The role of tumor stem-cell in regrowth of the tumor following drastic applications. Acta Unio Int Contra Cancrum.

[10] Reya T, Clevers H (2005). Wnt signalling in stem cells and cancer. Nature.

[11] Jordan CT (2004). Cancer stem cell biology: from leukemia to solid tumors. Curr Opin Cell Biol.

[12] Pathak S (2002). Organ- and tissue-specific stem cells and carcinogenesis. Anticancer Res.

[13] Weigmann A, Corbeil D, Hellwig A (1997). Prominin, a novel microvilli-specific polytypic membrane protein of the apical surface of epithelial cells, is targeted to plasmalemmal protrusions of non-epithelial cells. Proc Natl Acad Sci USA.

[14] Yin AH, Miraglia S, Zanjani ED (1997). AC133, a novel marker for human hematopoietic stem and progenitor cells. Blood.

[15] Miraglia S, Godfrey W, Yin AH (1997). A novel five-transmembrane hematopoietic stem cell antigen: isolation, characterization, and molecular cloning. Blood.

[16] Eramo A, Lotti F, Sette G (2008). Identification and expansion of the tumorigenic lung cancer stem cell population. Cell Death Differ.

[17] Ricci-Vitiani L, Lombardi DG, Pilozzi E (2007). Identi?cation and expansion of human colon-cancer-initiating cells. Nature.

[18] Bertolini G, Roz L, Perego P (2009). Highly tumorigenic lung cancer CD133+ cells display stem-like features and are spared by cisplatin treatment. Proc Natl Acad Sci USA.

[19] Chen YC, Hsu HS, Chen YW (2008). Oct-4 expression maintained cancer stem-like properties in lung cancer-derived CD133-positive cells. PLoS One.

[20] Ling TY, Kuo MD, Li CL (2006). Identification of pulmonary Oct-4+ stem/progenitor cells and demonstration of their susceptibility to SARS coronavirus (SARS-CoV) infection *in vitro*. Proc Natl Acad Sci USA.

[21] Rosner MH, Vigano MA, Ozato K (1990). A POU-domain transcription factor in early stem cells and germ cells of the mammalian embryo. Nature.

[22] Burdon T, Smith A, Savatier P (2002). Signalling, cell cycle and pluripotency in embryonic stem cells. Trends Cell Biol.

[23] Doyle LA, Yang W, Abruzzo LV (1998). A multidrug resistance transporter from human MCF-7 breast cancer cells. Proc Natl Acad Sci USA.

[24] Woo T, Okudela K, Mitsui H (2010). Prognostic value of CD133 expression in stage Ⅰ lung adenocarcinomas. Int J Clin Exp Pathol.

[25] Salnikov AV, Gladkich J, Moldenhauer G (2010). CD133 is indicative for a resistance phenotype but does not represent a prognostic marker for survival of non-small cell lung cancer patients. Int J Cancer.

[26] Gabriella F, Marco P, Giuseppina B (2009). Targeting CD133 antigen in cancer. Expert Opin Ther Target.

[27] Corbeil D, Roper K, Hellwig A (2000). The human AC133 hematopoietic stem cell antigen is also expressed in epithelial cells and targeted to plasma membrane protrusions. J Biol Chem.

[28] Florek M, Haase M, Marzesco AM (2005). Prominin-1/CD133, a neural and hematopoietic stem cell marker, is expressed in adult human differentiated cells and certain types of kidney cancer. Cell Tissue Res.

[29] Shmelkov SV, Jun L, St Clair R (2004). Alternative promoters regulate transcription of the gene that encodes stem cell surface protein AC133. Blood.

[30] Yu Y, Flint A, Dvorin EL (2002). AC133-2, a novel isoform of human AC133 stem cell antigen. J Biol Chem.

[31] Fargeas CA, Joester A, Missol-Kolka E (2004). Identification of novel prominin-1/CD133 splice variants with alternative C-termini and their expression in epididymis and testis. J Cell Sci.

[32] Potgens AJ, Schmitz U, Kaufmann P (2002). Monoclonal antibody CD133-2(AC141) against hematopoietic stem cell antigen CD133 shows cross-reactivity with cytokeratin 18. J Histochem Cytochem.

[33] Corbeil D, Roper K, Fargeas CA (2001). Prominin: a story of cholesterol, plasma membrane protrusions and human pathology. Traffic.

[34] Bauer N, Fonseca AV, Florek M (2008). New insights into the cell biology of hematopoietic progenitors by studying prominin-1 (CD133). Cells Tissues Organs.

[35] Maw MA, Corbeil D, Koch J (2000). A frameshift mutation in prominin (mouse)-like 1 causes human retinal degeneration. Hum Mol Genet.

[36] Joo KM, Kim SY, Jin X (2008). Clinical and biological implications of CD133-positive and CD133-negative cells in glioblastomas. Lab Invest.

[37] Beier CP, Beier D (2011). CD133 negative cancer stem cells in glioblastoma. Front Biosci (Elite ED).

[38] Wang J, Sakariassen PO, Tsinkalovsky O (2008). CD133 negative glioma cells form tumors in nude rats and give rise to CD133 positive cells. Int J Cancer.

[39] Gunther HS, Schmidt NO, Phillips HS (2008). Glioblastoma-derived stem cel1-enriched cultures form distinct subgroups according to molecular and phenotypic criteria. Oncogene.

[40] Dong QG, Yao M, Geng Q (2008). Isolation and identification of human lung adenocarcinoma stem cells. Tumor.

[41] Meng X, Li M, Wang X (2009). Both CD133+ and CD133- subpopulations of A549 and H446 cells contain cancer-initiating cells. Cancer Sci.

[42] Hashimoto O, Shimizu K, Semba S (2011). Hypoxia induces yumor aggressiveness and the expansion of CD133-positive cells in a hypoxia-inducible factor-1α-dependent manner in pancreatic cancer cells. Pathobiology.

[43] Liu Q, Nguyen DH, Dong Q (2009). Molecular properties of CD133+ glioblastoma stem cells derived from treatment-refractory recurrent brain tumors. J Neurooncol.

[44] Sakariassen PO, Immervoll H, Chekenya M (2007). Cancer stem cells as mediators of treatment resistance in brain tumors: status and controversies. Neoplasia.

[45] Clement V, Dutoit V, Marino D (2009). Limits of CD133 as a marker of glioma serf-renewing cells. Int J Cancer.

